# Baicalein-Loaded Chitosan Films for Local Treatment of Oral Infections

**DOI:** 10.3390/polym17162167

**Published:** 2025-08-08

**Authors:** Magdalena Paczkowska-Walendowska, Anna Rył, Jakub Kwiatek, Natalia Rosiak, Kamil Szarzyński, Weronika Wawrzyniak, Julia Ziółkowska, Weronika Kuderska, Kaja Kręcka, Anna Marciniak, Tomasz M. Karpiński, Tomasz Plech, Andrzej Miklaszewski, Piotr Owczarz, Judyta Cielecka-Piontek

**Affiliations:** 1 Department of Pharmacognosy and Biomaterials, Poznan University of Medical Sciences, Rokietnicka 3, 60-806 Poznan, Poland; nrosiak@ump.edu.pl (N.R.); kamil.szarzynski@interia.pl (K.S.); we.wawrzyniak@gmail.com (W.W.); 86781@student.ump.edu.pl (J.Z.); weronika.kuderska@wp.pl (W.K.); kaja.krecka@onet.pl (K.K.); ania.marciniak02@gmail.com (A.M.); jpiontek@ump.edu.pl (J.C.-P.); 2 Science-Bridge Sp. z o.o., Chociszewskiego 24/8, 60-258 Poznan, Poland; 3 Department of Chemical and Molecular Engineering, Lodz University of Technology, Wolczanska 213, 93-005 Lodz, Poland; anna.ryl@p.lodz.pl (A.R.); piotr.owczarz@p.lodz.pl (P.O.); 4 Kwiatek Dental Clinic Sp. z o.o., Kordeckiego 22, 60-144 Poznan, Poland; jakubkwiatek@klinikakwiatek.pl; 5 Department of Pharmacology and Phytochemistry, Institute of Natural Fibres and Medicinal Plants—National Research Institute, Kolejowa 2, 62-064 Plewiska, Poland; 6 Department of Medical Microbiology, Medical Faculty, Poznan University of Medical Sciences, Rokietnicka 10, 60-806 Poznan, Poland; tkarpin@ump.edu.pl; 7 Department of Pharmacology, Medical University of Lublin, Radziwillowska 11, 20-080 Lublin, Poland; tomasz.plech@umlub.pl; 8 Faculty of Materials Engineering and Technical Physics, Institute of Materials Science and Engineering, Poznan University of Technology, 60-965 Poznan, Poland; andrzej.miklaszewski@put.poznan.pl

**Keywords:** baicalein, chitosan films, oral infections, drug delivery, biomaterials, regenerative dentistry, biocompatibility

## Abstract

Oral infections and tissue defects remain significant clinical challenges, often requiring localized, sustained, and multifunctional therapeutic solutions. In this study, baicalein-loaded chitosan films were developed and comprehensively characterized as novel biomaterials for oral and maxillofacial applications. Using a 3^2^ factorial design, nine film formulations were prepared via solvent casting, varying chitosan molecular weight and composition. Physicochemical and structural analyses (microscopy, SEM, FTIR, and XRPD) confirmed uniform drug distribution and matrix compatibility. Mechanical testing and dissolution studies demonstrated zero-order baicalein release kinetics, with controlled, sustained delivery influenced by chitosan content and molecular weight. The optimal formulation (F5: CS MMW 2%, Gel 2%) combined favorable mechanical integrity, drug release, and potent antioxidant and anti-inflammatory activities. Further evaluation on 3D anatomical models simulating bone and soft tissue defects highlighted excellent membrane adaptability, stability, and ease of handling under conditions mimicking clinical surgery. The films acted as effective barriers in guided tissue regeneration and donor site protection, with improved surgical visibility due to their baicalein-induced coloration. Biocompatibility assays confirmed the safety of the materials, while antibacterial testing demonstrated activity against *Streptococcus mutans*. These results support the potential of baicalein-loaded chitosan films as multifunctional membranes for regenerative dentistry, periodontal therapy, and peri-implant care. The modular formulation design provides a platform for future integration of additional bioactive agents, paving the way for personalized, advanced wound healing solutions.

## 1. Introduction

Oral infections, including periodontitis, peri-implantitis, and post-extraction wound infections, remain a major clinical concern due to the complex microbial environment of the oral cavity and the limitations of traditional treatments [1]. Topical or systemic antibiotics often fail to provide sustained therapeutic levels at the site of infection and may contribute to resistance development or systemic side effects [2]. Consequently, there is a growing need for localized drug delivery systems that offer controlled release, mucosal adhesion, and multifunctional bioactivity—ideally with anti-inflammatory, antioxidant, and antimicrobial effects.

Among the candidates for such therapeutic applications, baicalein (5,6,7-trihydroxyflavone) stands out as a natural compound with a broad pharmacological profile. Baicalein is a major bioactive flavonoid found in the roots of *Scutellaria baicalensis* Georgi, commonly known as skullcap [3]. This traditional medicinal plant has been used in East Asian medicine for centuries, primarily for the treatment of inflammatory diseases, respiratory conditions, and infections [4]. Modern studies confirm that baicalein possesses potent anti-inflammatory, antioxidant and antimicrobial [5]. It has been shown to inhibit pro-inflammatory cytokines, scavenge reactive oxygen species (ROS), and suppress microbial growth—including oral pathogens such as *Streptococcus mutans*.

Despite its therapeutic promise, baicalein suffers from poor aqueous solubility, low bioavailability, and instability under physiological conditions, which limit its clinical application, especially in topical formulations. Incorporating baicalein into a biocompatible polymeric matrix may overcome these limitations by stabilizing the compound, enhancing its retention at the target site, and providing controlled release.

Various systems have been explored for oral and periodontal applications, including hydrogels, nanofibers, microspheres, and liposomal carriers, each differing in drug release kinetics, mucoadhesiveness, and biocompatibility [6,7,8]. Chitosan-based platforms are also well documented, often formulated as gels, suspensions, or structures requiring chemical crosslinking [9,10]. Chitosan (CS), a natural cationic polysaccharide derived from chitin, has emerged as an ideal carrier for such applications. It exhibits biodegradability, mucoadhesiveness, non-toxicity, and inherent antimicrobial activity, making it particularly suitable for use in oral environments [11]. Chitosan-based films are especially attractive as local drug delivery platforms due to their thin, flexible structure, high surface area, and ability to conform to moist oral tissues. When blended with gelatin, a protein with good mechanical and biocompatible properties, chitosan films can form stable, elastic matrices that enhance drug entrapment and tissue compatibility [12]. Chitosan–gelatin systems are widely studied in the literature due to the possible formation of complexes between the two biopolymers due to the electrostatic interactions between the protonated aminated groups of chitosan and the negatively charged carboxyl groups of gelatin [13].

The solution casting method is commonly used for preparing such films. It involves dissolving chitosan and gelatin in an acidic aqueous solution, incorporating the active substance (e.g., baicalein), and pouring the mixture into molds for drying. This process allows fine control over film thickness, drug loading, and surface morphology, which directly affect release kinetics, mechanical strength, and biological performance [14]. Unlike semi-fluid systems, the film exhibits mechanical stability, ease of surgical handling, and adaptability to complex anatomical contours, which are critical in oral wound care and regenerative procedures [15]. The integration of multiple therapeutic functions in a structurally stable and clinically applicable membrane highlights the innovative character of such formulation compared to existing delivery modalities. In regenerative dentistry, bioactive films are used as scaffolds or barriers in guided tissue regeneration (GTR) and guided bone regeneration (GBR), promoting healing and tissue integration [16]. Despite promising in vitro and in vivo results, clinical translation is still ongoing. Challenges such as optimizing mechanical properties, ensuring long-term stability, and regulatory approval remain areas of active research.

In this study, we aimed to develop and optimize baicalein-loaded chitosan–gelatin films for potential application in oral infections and surgical wound care. Our work is distinct in its use of a systematic 3^2^ factorial design to investigate the synergistic effects of chitosan molecular weight and chitosan–gelatin ratios on film performance. The resulting films were comprehensively evaluated for physicochemical properties, mechanical strength, drug release kinetics, and a broad range of biological activities, including antioxidant, anti-inflammatory, antimicrobial, and cytocompatibility assays.

By identifying an optimized formulation with balanced therapeutic and material properties, this work advances the field by offering a multifunctional, biopolymer-based platform tailored for targeted oral therapy, with potential applications in mucosal and periodontal drug delivery, differentiating it from prior studies that focused primarily on either baicalein or chitosan alone, or lacked integrated optimization and multi-parameter evaluation.

## 2. Materials and Methods

### 2.1. Chemicals

Baicalein 99% (5,6,7-Trihydroxyflavone, BAI) was obtained from TriMen Chemicals Sp. z o.o. (Łódź, Poland). Excipients, such as chitosans (low molecular weight = LMW 20–300 cps, 1% in 1% acetic acid; medium molecular weight = MMW 200–800 cps; high molecular weight = HMW 800–2000 cps), gelatin (Gel), and glycerol were supplied from Sigma-Aldrich (Poznan, Poland). Reagents for activity assays (2,2-Diphenyl-1-picrylhydrazyl (DPPH), sodium chloride, bovine serum, hexadecyltrimethylammonium bromide (CTAB), hyaluronic acid (HA), dissolution studies (phosphate buffer), and bioadhesive tests (mucin from porcine stomach) were obtained from Sigma-Aldrich (Poznan, Poland). The HPLC grade acetonitrile and water were obtained from Merck (Darmstadt, Germany). High-quality pure water and ultra-high-quality pure water were prepared using a Direct-Q 3 UV Merck Millipore purification system.

### 2.2. Preparation of Baicalein-Loaded Chitosan Films

The films were prepared using the solution casting method. Based on the design of experiments (DoE) approach and a 3^2^ factorial design (Statistica 13.3 software, TIBCO Software Inc., Palo Alto, CA, USA), the composition of the films (chitosan molar mass, CS MW: LMW marked as 100, MMW as 600, and HMW as 1100; amount of chitosan, CS %; amount of gelatin, Gel %) was selected and presented in Table 1.

A volume of 20 mL was added to a beaker, which was then heated to 60 °C. Gelatin was subsequently dissolved under continuous stirring using a magnetic stirrer. Then baicalein (constant mass 200 mg), glycerol (constant volume 0.1 mL), chitosan (in the appropriate amount according to Table 1), and acetic acid (constant volume 0.2 mL) was added and stirred for 30 min. The ready hydrogel was poured into a Petri dish and left for 48 h to evaporate the solvent.

The Young modulus, plasticity, release of BAI, antioxidant activity (as measured by the DPPH method), and anti-inflammatory activity (as indicated by the degree of hyaluronidase enzyme inhibition) were the parameters used to evaluate the effectiveness of chitosan films.

#### 2.2.1. Microscopic Analysis

The surface of the films and the 3D image were analyzed using a Keyence VHX-S770E digital microscope (Keyence International, Mechelen, Belgium).

#### 2.2.2. Scanning Electron Microscopy (SEM)

A scanning electron microscope (Quanta 250 FEG, FEI, Waltham, MA, USA) was used to create SEM pictures to evaluate the manufactured films’ morphology. The films were sputter-coated with gold prior to examination.

#### 2.2.3. X-Ray Powder Diffraction (XRPD)

The created films were evaluated in a crystallographic assay using a copper anode (CuKα—1.54 Å) and X-ray diffraction equipment (Panalytical Empyrean, Almelo, The Netherlands). The measurements were conducted using the Bragg–Brentano reflection mode arrangement, with 40 mA and 45 kV as the parameters. The measurement range was determined to be between 3° and 60° with a step size of 0.05° and a measurement time of 45 s per step.

#### 2.2.4. Fourier Transform Infrared Spectroscopy (FTIR)

FTIR spectra were acquired in absorbance mode, spanning 400 to 4000 cm^−1^, using an IRTracer-100 (Shimadzu, Kyoto, Japan) spectrophotometer and LabSolutions IR software (version 1.86 SP2, Shimadzu, Kyoto, Japan). The spectrometer was set up with a resolution of 4 cm^−1^, 400 scans, and Happ–Genzel apodization.

#### 2.2.5. Molecular Modeling

The baicalein (BAI) 3D structure (CID: 5281605) and chitosan (Cs) 2D structure (CID: 129662530) in sdf format was obtained from PubChem (https://pubchem.ncbi.nlm.nih.gov, accessed on 23 July 2025). A rough 3D geometry of Cs was generated using Avogadro 1.2.0 [17]. Gaussview 6.0.16 (Wallingford, CT, USA) was employed to draw gelatin structures. Gaussian 16C (Wallingford, CT, USA) software was utilized to optimize the BAI and Cs geometries (B3LYP/6-31 (d,p) level) prior to molecular modeling. Gaussian 16C took advantage of computational resources made available specifically via the service of “HPC cluster ‘Eagle’ for researchers,” provided through the Poznan Supercomputing and Networking Center (https://pcss.plcloud.pl, accessed on 23 July 2025).

Molecular modeling was performed using AutoDock 4.2.6 (website: http://autodock.scripps.edu, accessed on 24 July 2025). The process was divided into two stages: (1) preparation of the Cs–Gel complex, (2) preparation of the Cs–Gel–BAI complex. In stage 1, the Cs molecule (in mol2 format) was loaded using the AutoDock Tools’ ligand menu, whereas in stage 2, it was BAI. Next, the torsion tree was defined by choosing the root; the number of rotatable bonds was identified, and the file was saved in PDBQT format (Cs.pdbqt and Bai.pdbqt, respectively). As macromolecule (in PDB format) was imported in stage 1, Gel, while in stage 2, Cs–Gel complex. Polar hydrogen atoms were added; the Kollman and Gasteiger charges were computed for the macromolecules. Next, the file was saved in PDBQT format for a further simulation process. Grid spacing was set to default, whereas the center grid box values were position of macromolecule. The number of grid points along the x, y, and z dimensions were set as 55 × 55 × 55. The total grid points per map was 64,000. The output was saved in the grid parameter file (GPF) file format. After generating the GPF files, AutoGrid was run using them as input, producing corresponding grid log files (GLG format). Once AutoGrid completed successfully, the genetic algorithm was configured with default parameters. The Lamarckian genetic algorithm was applied, and the settings were saved in a docking parameter file (DPF). AutoDock was then executed using both the AutoDock executable and the DPF file as inputs, which resulted in a docking log file (DLG). This file contained detailed information, including the top ten binding energies and RMSD values describing the interaction between ligand and macromolecule. The results were further examined through AutoDock Tools’ analysis menu, with docking poses ranked by binding energy. The pose with the lowest binding energy was exported in PDBQT format. The conversion from PDBQT to PDB format was performed using Open Babel software (the Open Babel Package, version 2.4.0, http://openbabel.org, accessed on 25 July 2025). Final high-resolution molecular visualizations were created with Discovery Studio Visualizer 2025 v25.1.0.24284 (BIOVIA, Dassault Systèmes, San Diego, CA, USA, https://discover.3ds.com/discovery-studio-visualizer-download, accessed on 25 July 2025).

### 2.3. Characteristics of Chitosan Films

#### 2.3.1. Rheology

Mechanical characterization was carried out using a Physica MCR 502 rotational rheometer (Anton Paar, Graz, Austria) equipped with a Universal Extensional Fixture (UXF). Strain-controlled extensional measurements at room temperature were carried out at different strain rates: 0.005, 0.02, and 0.05 s^−1^. The set parameters reflected static and dynamic loads, respectively. Each experiment was performed in triplicate.

#### 2.3.2. Swelling Index and Dissolution

Each film was individually weighed and placed in a 25 mL beaker that contained 10 mL of an artificial saliva solution at pH of 6.8 and at 37 ± 0.5 °C. The films were taken out, cleaned with filter paper, and reweighed at the preset time intervals (15, 30, 60, 120, and 240 min). The swelling index was calculated by using the following formula:

$$SI=\frac{W_{2}-W_{1}}{W_{1}}$$

where SI is the swelling index, $W_{1}$ is the initial weight of the film, and $W_{2}$ is the weight of the film after the swelling time interval. Each experiment was performed in triplicate.

Using an Agilent 708-DS dissolving device (Agilent, Santa Clara, CA, USA), the dissolution properties of films were investigated. The conventional basket method was employed, with 200 mL of artificial saliva solution serving as the acceptor solution and stirring at 37 ± 0.5 °C and 50 rpm. The same volume of temperature-adjusted medium was added to liquid samples that were collected at prearranged intervals. A nylon membrane filter with a diameter of 0.45 μm was used to filter the samples. The quantities of baicalein in the acceptor solutions were ascertained using the previously mentioned HPLC procedure [5]. Sink conditions were maintained throughout the study. The study was repeated six times.

#### 2.3.3. Biological Activity

Antioxidant Activity

2,2-Diphenyl-1-picrylhydrazyl (DPPH) was used in an experiment to measure the antioxidant activity, according to the previously described protocol [18]. The study was repeated nine times.

Anti-Hyaluronidase Activity

Anti-inflammatory activity was tested as a possibility of inhibiting the activity of the hyaluronidase enzyme, according to the previously described protocol [18]. The study was repeated nine times.

#### 2.3.4. Microbiological Activity

The inhibitory potential of the growth of microorganisms in the studied systems was determined using the disk diffusion method on agar, determining the growth inhibition zones [19]. The *Streptococcus mutans* ATCC strain was used in the investigation.

#### 2.3.5. Biocompatibility Assay

The MTT test was used to assess the vitality of human normal skin fibroblasts (Hs27 cells) cultured on film for 24 h [20].

#### 2.3.6. Blood-Induced Behavioral Testing

Venous blood was collected into standard collection tubes containing EDTA as an anticoagulant (purple cap tubes).

Pre-weighed and labeled test tubes containing the material were prepared for testing. Using a syringe equipped with a needle, 1 mL of the EDTA-stabilized blood was carefully introduced into each test tube. To prevent uncontrolled or uneven absorption at this stage, the membrane was deliberately positioned away from the incoming blood during filling. After the blood was added, the membrane was placed into each test tube and allowed to soak for 5 min. Following this period, the membrane was removed and evaluated for changes in structure, surface properties, and its ability to conform to anatomical shapes.

#### 2.3.7. iPRF-Induced (Injectable Platelet-Rich Fibrin) Behavioral Testing

Venous blood was processed in a dedicated tube for iPRF preparation (green iPRF tube). The blood was centrifuged using a device designed for this purpose (centrifugation time: 14 min, relative centrifugal force (RCF): 207) to obtain the plasma fraction in the form of injectable platelet-rich fibrin (iPRF).

Pre-labeled and pre-weighed test tubes containing the test material were prepared. Using a syringe with a needle, 1 mL of the iPRF fraction was carefully deposited into each test tube. Importantly, during the addition of iPRF, the membrane was intentionally displaced within the tube to prevent immediate contact with the liquid at this stage, thereby avoiding uncontrolled soaking during filling. The film was then immersed in the iPRF for 5 min. After this soaking period, the material was removed from the test tube and assessed for its structural behavior, surface properties, and ability to conform to anatomical contours.

#### 2.3.8. Evaluation on a 3D Anatomical Model with Bone Defect and Model of Soft Tissue

Evaluation on a 3D anatomical model with bone defect: For the study, pre-labeled and pre-weighed test tubes containing the test material were prepared. Venous blood was collected from a patient using a cannula directly into a collection tube containing an anticoagulant (tube with a purple cap). Using a syringe with a needle, 1 mL of the collected blood was carefully deposited into each of the smaller test tubes containing the film. Importantly, during the addition of blood, the film was deliberately displaced within the tube to prevent immediate contact with the blood at this stage. This step was performed to avoid uncontrolled soaking of the material during the filling process. The film was then soaked in the blood for 5 min. After the soaking period, the membrane was removed from the test tube and assessed for its surface properties, adherence capability, and ability to conform to anatomical contours.

Evaluation on a 3D anatomical model of soft tissue: The membrane was placed in a pre-weighed and labeled test tube prior to the procedure. Subsequently, 1 mL of 0.9% sodium chloride solution was carefully added to the test tube, in a volume sufficient to ensure complete immersion of the membrane. During the addition of the solution, the membrane was gently displaced to prevent it from being directly exposed to the saline stream, thus avoiding localized oversoaking or potential surface damage. The membrane was soaked for 5 min. After the soaking period, the material was removed from the solution and assessed for structural changes and its ability to adapt to anatomical contours.

### 2.4. Statistical Analysis

Statistica 13.3 was used to do the statistical analysis. The variations between the mean values were examined using Duncan’s post hoc test, Tukey’s post hoc range test for multiple comparisons, and the ANOVA test. Group differences were considered significant at *p* < 0.05. Correlations were examined using principal component analysis (PCA) and PQStat software version 1.8.4.142 (2022).

## 3. Results and Discussion

Nine baicalein-loaded chitosan films (F1–F9) were successfully prepared using the solvent casting method based on a factorial design. Each film exhibited distinct physicochemical and biological properties depending on the chitosan molecular weight (LMW, MMW, and HMW) and the ratio of chitosan to gelatin (according to Table 1). An important parameter is the influence of film mass (related to thickness and total polymer content) on the mechanical performance and drug release behavior. The mass increased with the increase in polymer concentration in the system. The mass of the film can directly affect its structural stability, flexibility, swelling behavior, and diffusion-controlled drug release [21], which will be discussed later in the work.

**Figure 1:**
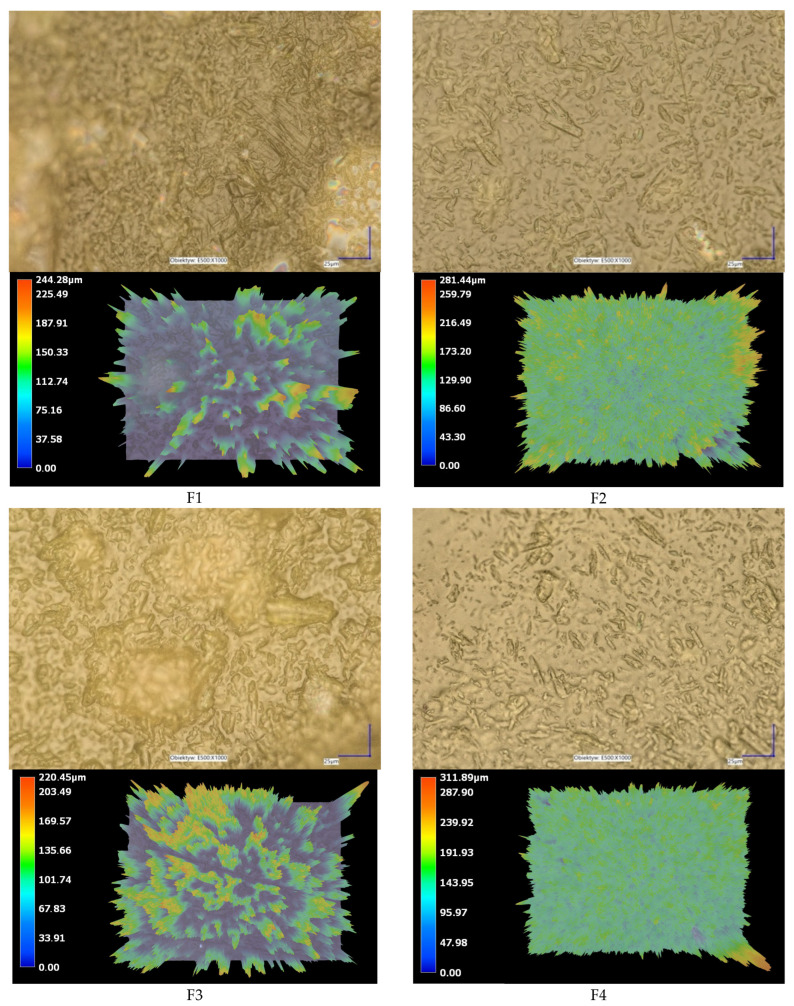

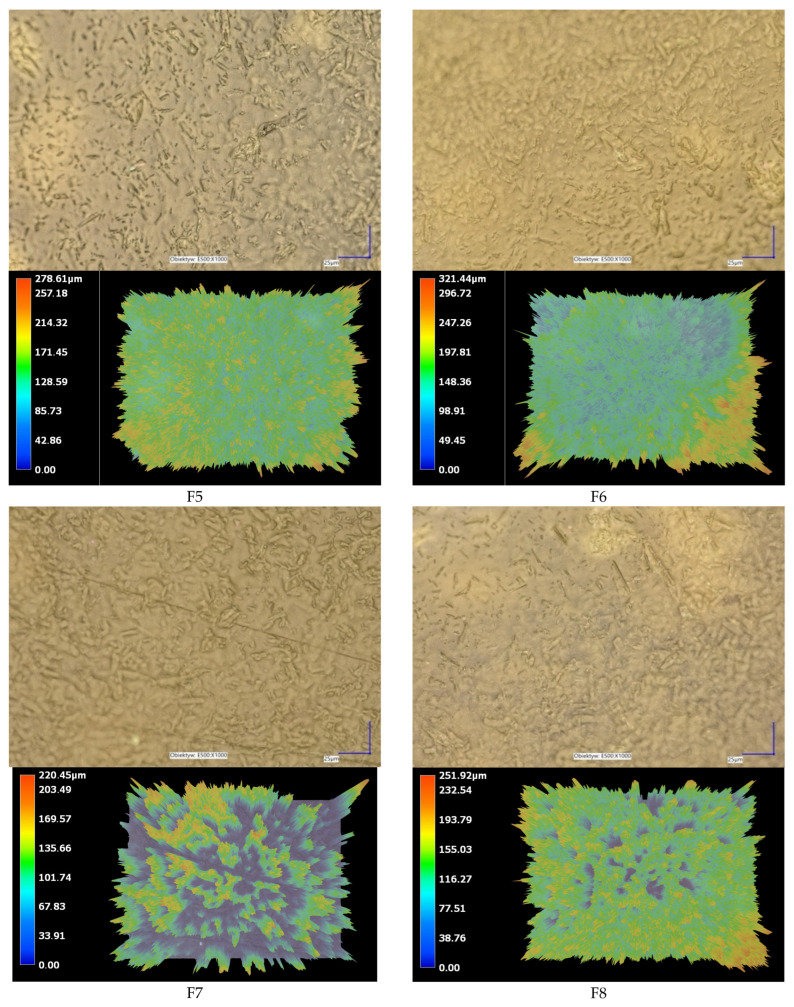

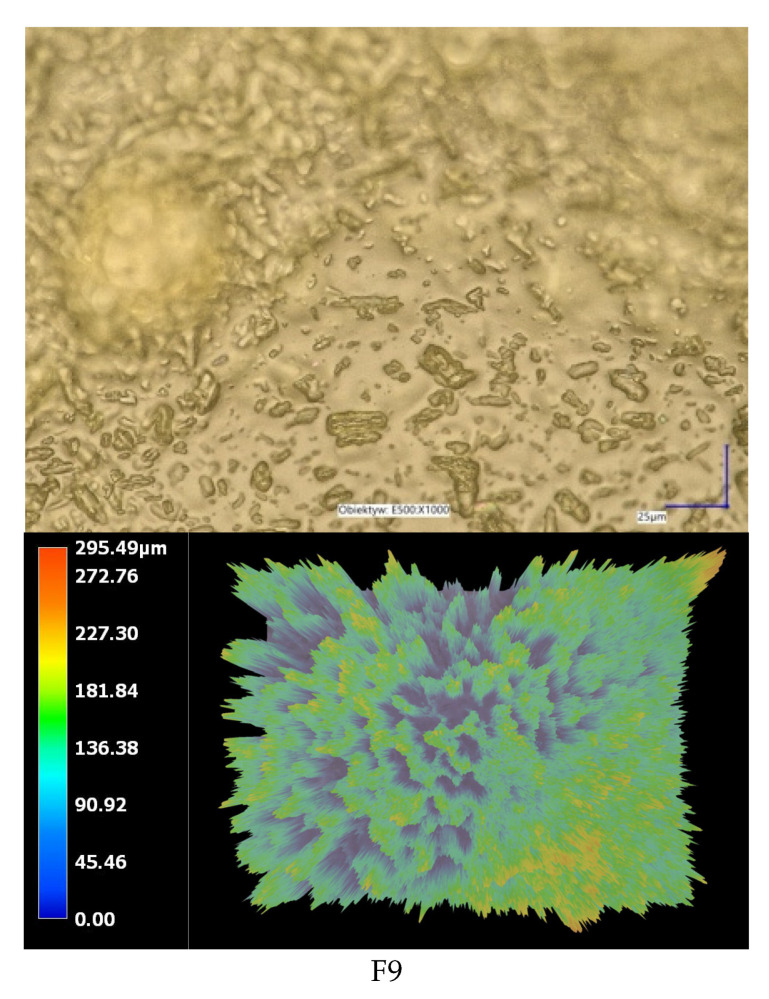
Photographic images of the surface morphology of films F1–F9. The visual appearance of each film is shown to highlight differences in texture, uniformity, and transparency resulting from variations in formulation composition.

**Figure 2:**
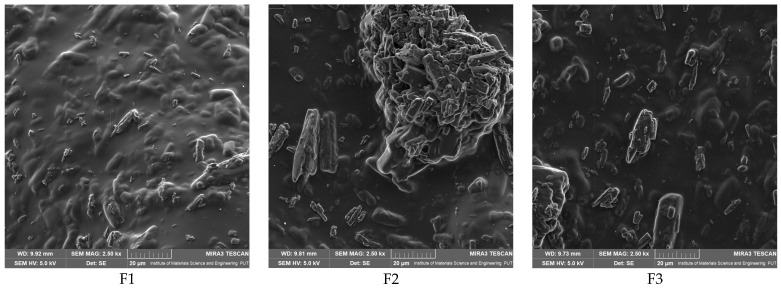

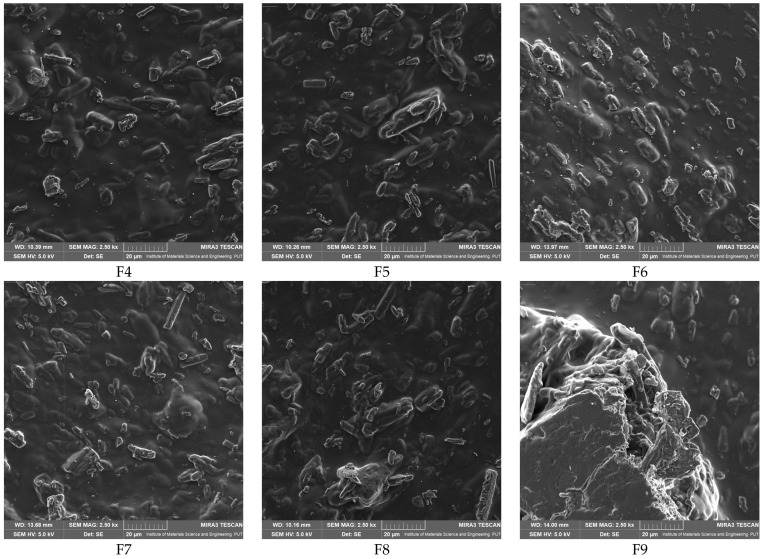
Scanning electron microscopy (SEM) images of the surface structure of films F1–F9. The micrographs reveal surface topography and microstructural features influenced by the type and concentration of film-forming components.

Microscopic (Figure 1) and SEM (Figure 2) imaging demonstrated that increasing chitosan molecular weight and gelatin content influenced film surface structure, with smoother, more homogeneous textures observed for MMW-based systems. The particles of baicalein visible in microscopic and SEM images indicate a uniform distribution of the active substance within the membrane structure for most of the studied systems. In films containing CS MMW (F4–F6), the surface was smooth and homogeneous, without evident signs of agglomeration or component segregation, suggesting good compatibility between chitosan, gelatin, and baicalein. The small, dispersed baicalein particles observed in the structure are evenly distributed, with no tendency toward crystallization or cluster formation, confirming the effectiveness of the applied method for incorporating the active substance and homogenizing the system. In films F1–F3, containing CS LMW, slight surface irregularities and localized concentrations of the solid phase were noticeable, yet without clear segregation of baicalein, which also confirms its relatively uniform distribution. Conversely, in films F7–F9, based on CS HMW, a somewhat rougher and more fibrous structure was observed, which may result from increased system viscosity during film formation. Nevertheless, in this group as well, baicalein occurs as fine, well-dispersed particles, without major agglomeration spots. Moreover, 3D images confirm the uniform thickness of the obtained materials, indicating a reproducible material fabrication process (Figure 1). Such homogeneous distribution of the active substance across all films is crucial from the perspective of predictable drug release, bioactivity, and membrane stability. These observations are consistent with the results of baicalein release analyses, where systems F4–F6 exhibited the most stable and controlled kinetic profile.

**Figure 3:**
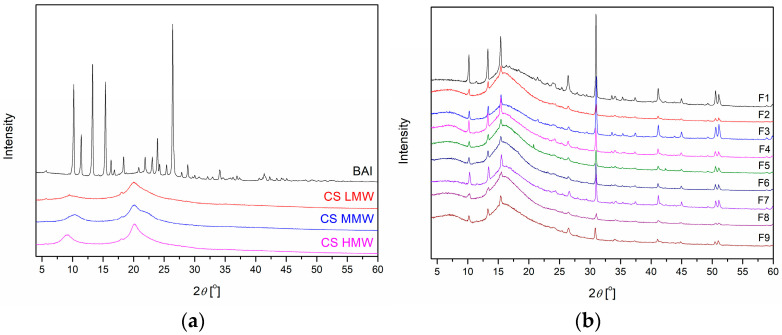
XRPD diffractograms of BAI and CS (**a**) and films F1–F9 (**b**).

The XRPD diffractograms presented in Figure 3 provide insight into the crystallographic structure of BAI, CS, and the BAI-loaded chitosan films (F1–F9). Pure baicalein exhibits a highly crystalline structure, as evidenced by the presence of numerous sharp and intense diffraction peaks within the 2θ range of approximately 10° to 30°. Notable reflections are observed at around 10.5°, 11.1°, 13.6°, 15.9°, and 27.6°, indicating the well-ordered molecular arrangement of the unprocessed compound [22]. In contrast, chitosan, depending on its molecular weight, displays a predominantly amorphous or semi-amorphous nature. Its XRPD pattern is characterized by a broad halo centered around 2θ ≈ 19.8°–20.5°, which is typical of amorphous polysaccharides. With increasing molecular weight, a slight intensification and shift of this broad peak are observed, reflecting a modest increase in structural organization [23].

The XRPD profiles of the prepared films (F1–F9) differ significantly from those of the individual components. Across all formulations, the characteristic sharp peaks of crystalline baicalein are markedly reduced or completely absent, indicating a substantial loss of crystallinity. This suggests that baicalein was successfully incorporated into the chitosan–gelatin matrix in an amorphous or nanocrystalline state. Films F1–F3 (based on CS LMW) still show faint residual peaks in the lower 2θ range (10°–15°), hinting at minor crystalline domains of baicalein. However, films F4–F6 (CS MMW) exhibit the most uniform and diffuse patterns, with no apparent crystalline signals, confirming the homogeneous amorphous dispersion of baicalein and optimal polymer compatibility. Films F7–F9 (CS HMW) also display broad, low-intensity signals with a subtle maximum near 2θ ≈ 22°, possibly attributed to partial ordering within the polymer network. The appearance at 31.2° peak in the films—despite being negligible in raw chitosan or baicalein—likely indicates the formation of a new, weakly crystalline domain due to polymer–drug interactions and structural reorganization during film formation. This is a common phenomenon in drug-loaded polymer systems, where the physical state of the components changes significantly compared to their raw forms.

Overall, the XRPD analysis confirms that the casting process effectively transformed crystalline baicalein into an amorphous state within the polymer matrix.

**Figure 4:**
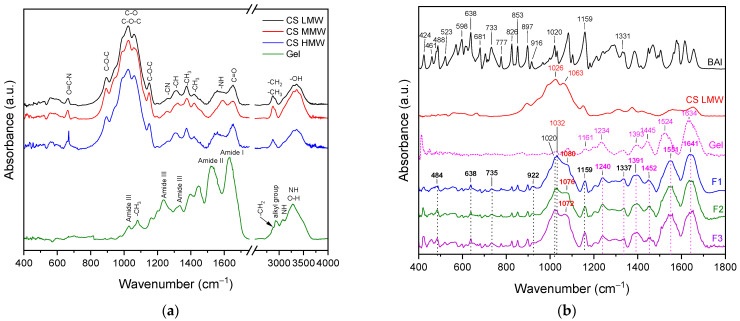

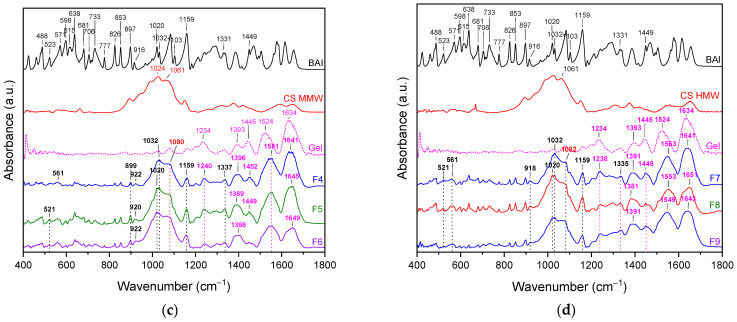
Spectra of CS and Gel (**a**), BAI and films 1–3 (**b**), 4–6 (**c**), and 7–9 (**d**).

Fourier transform infrared (FTIR) spectroscopy was employed to characterize BAI, Gel, CS LMW/MMW/HMW, and films F1–F9 (Figure 4).

The obtained spectra revealed several characteristic absorption bands corresponding to functional groups typical of chitosan. In the lower wavenumber region, bands observed at 663 cm^−1^ (LMW, MMW) and 669 cm^−1^ (HMW) were assigned to the vibration of the O=C-N group, indicative of residual amide functionalities [24]. Peak at 893 cm^−1^ is attributed to C-H bending vibrations, commonly associated with saccharide ring structures [25]. Bands observed at 1024 cm^−1^ and 1061 cm^−1^ correspond to C-O stretching vibrations within the polysaccharide backbone, confirming the integrity of glycosidic linkages in all molecular weight variants [26]. Additionally, the band at 1150 cm^−1^ was assigned to C-O-C bending vibration, characteristic of glycosidic bridges [25]. In the mid-infrared region, bands in the range of 1258–1261 cm^−1^ are attributed to C-N stretching vibrations [27], while signals at 1307–1321 cm^−1^ are assigned to Amide III (C-N stretching vibrations) [26]. Differences in the positions of the LMW/MMW/HMW bands may reflect variations in intra- and intermolecular interactions as a function of chain length. The peaks at 1375 cm^−1^ and 1420 cm^−1^ are attributed to antisymmetric and symmetric -CH_3_ deformations, respectively [27]. The Amide II band, associated with N-H bending and primary amine deformation (N-H bending vibration), is observed at 1558 cm^−1^ in the LMW and HMW samples while shifting to 1591 cm^−1^ in the MMW sample [27]. Band at 1653 cm^−1^ corresponds to C=O stretching vibrations of amide groups (Amide I), related to residual N-acetylated units in the polymer. The high-frequency region band observed at 2872 cm^−1^ was assigned to aliphatic C-H stretching vibrations, whereas a broad absorption band with maximum at 3356 cm^−1^ (observed in all samples) corresponds to O-H stretching vibrations [28].

Gel exhibited characteristic absorption bands corresponding to its polypeptide backbone and functional side chains. Skeletal C-O stretching vibrations (Amide III) are observed at 1030 cm^−1^ and 1080 cm^−1^ [29]. The band at 1234 cm^−1^ was attributed to a combination of C-N stretching and N-H bending vibrations, characteristic of Amide III [29]. Peak at 1333 cm^−1^ correspond to CH_2_ bending vibrations from proline side chains, while 1393 cm^−1^ corresponds to CH_2_ wagging vibrations [29,30,31]. It indicates Gel’s proline-rich composition. The deformation of -CH_2_ groups was also observed at 1445 cm^−1^ [31]. The Amide II region represents bands at 1524 cm^−1^ (N-H bending, C-N and C-C stretching vibrations) [29]. A strong absorption band at 1634 cm^−1^ is assigned to the Amide I region (mainly C=O stretching/hydrogen bond coupling with COO, with contributions from NH stretching vibration). It is reflecting the secondary structure and potential β-sheet content of the Gel [29,30,31,32].

FTIR absorption spectra of baicalein have main characteristic bands in the range of 400–1700 cm^−1^. The baicalein bands for which changes are observed in the F1–F9 spectra collect in Table 2.

In particular, the bands observed at specific wavenumbers (cm^−1^) correspond to the following vibrations: 424 (HCCC torsion—ring A), 461 (O-H—ring A), 488 (CCCH torsion—ring A, B, C and CCCO torsion—ring A, C), 523 (CCCH torsion—ring A and CCCO torsion—ring A, C), 571 (CCCH torsion—ring A and CCCO torsion—ring A, C), 598 (CCCH torsion—ring A–C), 615 (CCOH torsion—ring A and CCCC torsion—ring A, C), 638 (CCH bending—ring B) and COC bending—ring A–C)), 681 (CCC bending—ring A, B; CCO bending—ring A, C and CCH bending—ring B), 706 (CCC bending—ring A, C; CCO bending—ring A, C; CCH bending—ring A, C), 733 (C-H—ring B), 777 (CCC bending—ring A and CCO bending—ring A–C), 826 (C-H—ring B), 853 (C-H—ring A), 897 (O-H—ring A and C-H—ring C), 916 (deformation all molecule), 1020 (C-O-C—ring C), 1032 (C-C-C—ring B), 1159 (C-H—ring B),1331 (C=C—ring C, O-H—ring A and C-H—ring B), and 1449 (O-H—ring A) [33,34].

Changes (shifts of the peaks) were also observed in the bands characteristic of gelatin. The summary is presented in Table 3.

Literature indicates that due to the presence of numerous hydroxyl groups (-OH), Cs can create bonds with carboxylic groups (-OOH) and amine (-NH_2_) collagen [35]. In the acidic environment of carboxylic groups, negatively charged, they interact with ionic interactions with positively charged Cs amino groups. These interactions—including both side, final, and functional chains (carboxylic, amine, and hydroxyl)—leading to the formation of numerous complexes. This is in accordance with the results of FTIR analysis. The band of Gel observed at about 1634 cm^−1^ (ν_C=O_ of carboxylate anion + ν_NH_ (Amide I)) moves to higher wavenumber (see Table 3), while the band of CS LMW/MMW/HMW at 1061 cm^−1^ (C-O stretching vibrations within the polysaccharide backbone) moves to 1080 cm^−1^ (F1), 1076 cm^−1^ (F2), 1072 cm^−1^ (F3), 1080 cm^−1^ (F4–F6), and 1082 cm^−1^ (F7–F9). Next, shift the Amide II bands of gelatin (1524 cm^−1^) indicating the formation of hydrogen bonds between the -NH groups of Gel and C-O groups of Cs [36].

**Figure 5:**
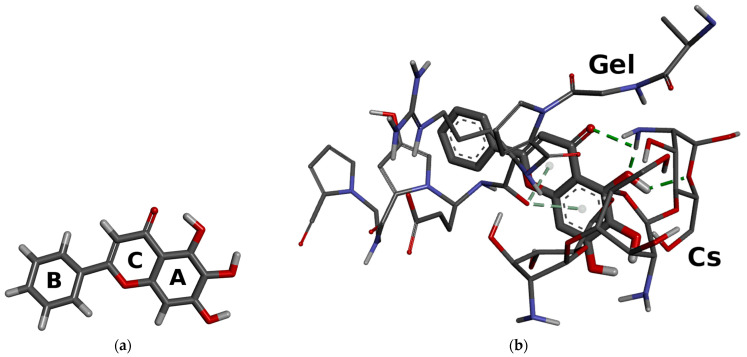
3D structure of baicalein (**a**) and visualization of the simple Cs–Gel–baicalein model (**b**). Legend: green dashed line—hydrogen bond, grey dashed line—non-covalent interactions (CO···π interaction).

Therefore, it can be concluded that the Cs–Gel complex is formed primarily through electrostatic interactions and hydrogen bonding between the two components. According to Qiao et al., in CS–Gel films, CS mainly interacts with the carboxyl groups of Gel via electrostatic forces [36]. In addition, based on the study by Chen et al. [37], who investigated Cs–Gel functional composite films loaded with flavonoids (naringenin, apigenin, and luteolin), the shifts observed in our spectra at 1524 cm^−1^ (Gel) and 1634 cm^−1^ (CS) in F1–F9 suggest the formation of intermolecular hydrogen bonds between baicalein and the CS–Gel backbone. Molecular modelling was employed to support the FTIR analysis and to predict the possible interactions within the CS–Gel–baicalein formulation. The 3D structure of baicalein is shown in Figure 5a, and the visualization of the simple CS–Gel–baicalein model is shown in Figure 5b. Similar approaches combining molecular modelling with FTIR spectroscopy have been previously reported in the literature [38,39,40,41], demonstrating their usefulness in identifying hydrogen bonding and other non-covalent interactions.

The visualization indicated the formation of non-covalent interactions (CO···π interaction) between the C-O group of Gel and aromatic rings (A and C) of baicalein. Based on this, the shifts of the baicalein bands observed at 523 cm^−1^ (CCCH torsion—ring A and CCCO torsion—ring A, C) and 571 cm^−1^ (CCCH torsion—ring A and CCCO torsion—ring A, C) suggest the involvement of aromatic rings A and B in non-covalent interactions, particularly hydrogen bonding. In addition, visualization suggests formation of hydrogen bonding interactions between (i) C=O group of baicalein and N-H group of Cs, (ii) C-O group (ring A) of baicalein and N-H group of Cs, and (iii) O-H group (ring A) of baicalein and C-O group of Cs.

The tensile tests conducted at three different deformation rates aimed to simulate a range of mechanical conditions that the films may encounter during their practical application (Table 4). Low deformation rates represent static or quasi-static conditions, which are typical for wound dressings or healing membranes that need to maintain integrity over extended periods without undergoing rapid or repetitive stretching. Under these conditions, materials with a lower Young’s modulus and moderate yield point are desirable, as they provide enough flexibility to conform to the wound site while still offering adequate mechanical support [42]. The results indicated that formulations F1 and F7 performed best under these static conditions, exhibiting the optimal balance of flexibility and strength required for post-extraction dressings and healing membranes. Their mechanical properties suggest good elasticity and sufficient resistance to deformation, which are essential for patient comfort and effective wound coverage.

In contrast, higher deformation (strain) rates (0.05 1/s) mimic dynamic loading scenarios such as movements involving repeated stretching, bending, or mechanical fatigue. These conditions demand materials with higher stiffness (Young’s modulus) and a higher yield stress point to withstand larger forces and prevent premature failure. The preparation F8 was identified as the most suitable under these dynamic conditions, demonstrating superior mechanical strength and resistance to deformation.

**Figure 6:**
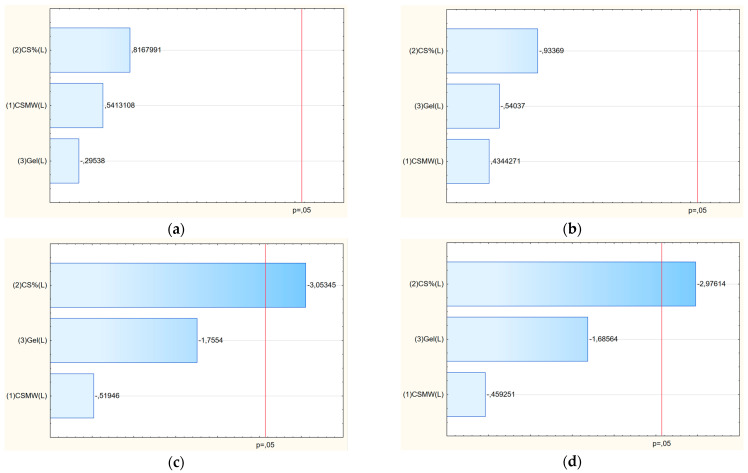
Pareto charts illustrating the standardized effects of formulation variables on Young’s modulus at strain rate 0.005 1/s (**a**) and 0.02 (**b**), and yield stress of films F1–F9 at rate 0.005 1/s (**c**) and 0.02 1/s (**d**). Significant factors are those extending beyond the reference line, indicating a statistically meaningful contribution to the mechanical properties.

Based on the Pareto chart analysis (Figure 6), it is evident that the yield stress is significantly influenced by the percentage of chitosan (CS) in the system; as the concentration of CS increases, the yield stress decreases. In the case of Young’s modulus, the effect strongly depends on the strain rate applied. The results for high strain rates appear particularly interesting due to the negative impact of chitosan concentration. In this case, adding more chitosan to a material can led to a decrease in both stiffness (Young’s modulus or elastic modulus) and yield strength. This may be due to the limited time for stress relaxation, leading to local microcracks or loss of cohesion. From a structural perspective, chitosan molecules can interfere with the polymer matrix network by disrupting strong intermolecular interactions—such as hydrogen bonding or ionic crosslinking—that contribute to the stiffness and mechanical integrity of the composite [43]. For example, in composites containing gelatin or other proteins, higher chitosan content may reduce the density of crosslinks, leading to a less compact and less rigid network. This structural disruption lowers the material’s Young’s modulus, which quantifies resistance to elastic deformation.

Yield strength, defined as the stress at which a material undergoes irreversible deformation, is similarly affected. Increased chitosan content typically introduces more flexible domains and reduces the cohesive forces within the matrix, making the material more prone to permanent deformation under lower applied stresses. Studies have shown that chitosan-based materials often exhibit lower mechanical strength compared to other polysaccharides or protein-based films, especially when not chemically crosslinked or reinforced [44,45]. Furthermore, chitosan’s molecular weight and degree of deacetylation influence its mechanical impact. Higher molecular weight chitosan can increase viscosity and form entangled networks, sometimes improving strength, but excessive amounts still tend to soften the overall composite. Lower degrees of deacetylation reduce intermolecular interactions, weakening mechanical properties further [46].

This distinction between low and high deformation rates highlights the importance of tailoring film properties to their intended clinical use. For applications requiring durability and resistance to mechanical stress, such as membranes subjected to frequent movement, formulations like F8 should be prioritized. Meanwhile, more flexible formulations like F1 and F7 are better suited for static or low-stress environments.

**Figure 7:**
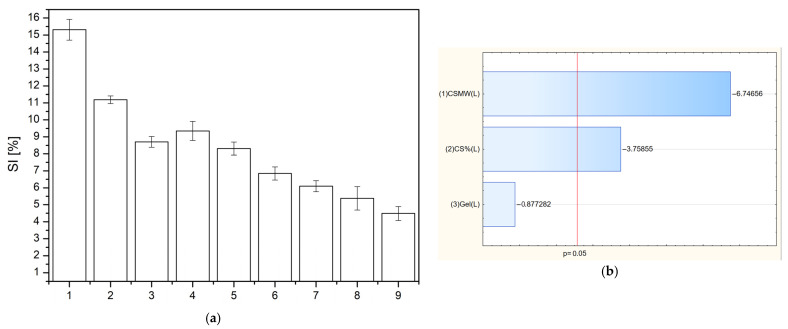
Swelling index of films F1–F9 in artificial saliva after 60 min (**a**) and Pareto chart showing the standardized effects of swelling index (**b**).

As shown in Figure 7, the swelling index (SI) increases with decreasing molecular weight of chitosan and decreasing chitosan concentration in the formulation. This behavior can be attributed to the structural and physicochemical properties of chitosan. Lower molecular weight chitosan possesses shorter polymer chains, which results in a more open network structure within the matrix. This looser network allows water molecules to more easily penetrate and interact with the hydrophilic groups (e.g., –OH and –NH_2_) of chitosan, leading to greater swelling [46]. Similarly, reducing the chitosan concentration decreases the overall polymer density within the matrix. A lower polymer content results in less chain entanglement and crosslinking, thereby increasing the free volume available for water uptake. This facilitates greater diffusion of water into the matrix and enhances the swelling capacity.

**Figure 8:**
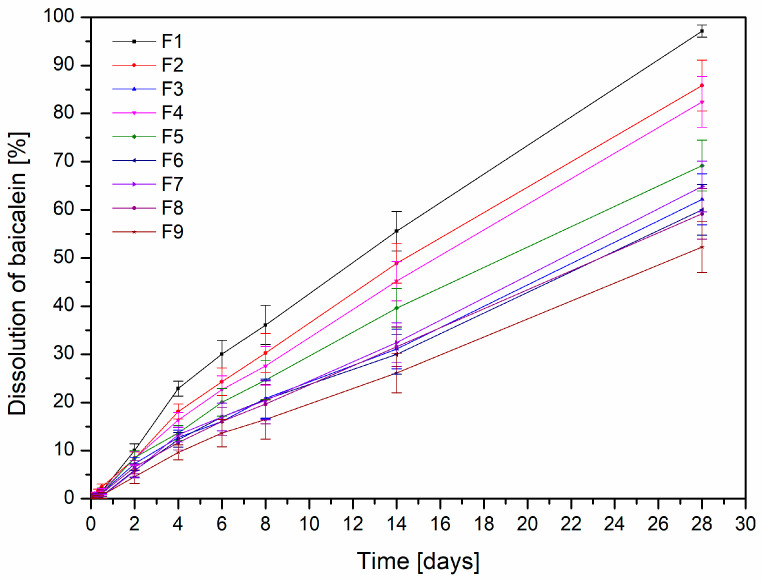
Release rate profiles of baicalein from films F1–F9.

**Figure 9:**
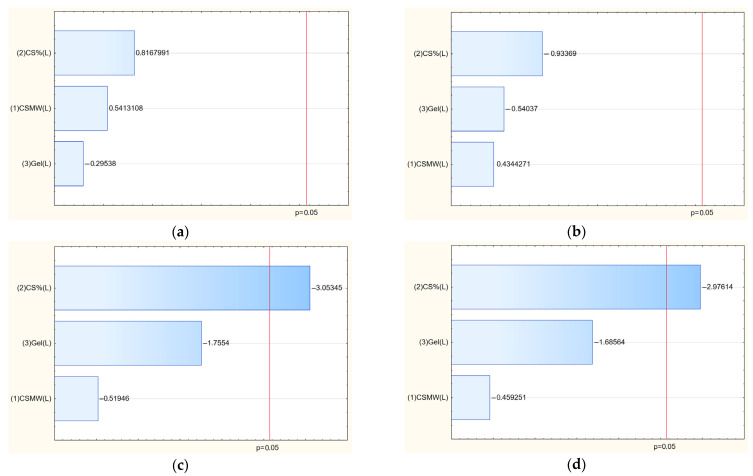
Pareto chart illustrating the standardized effects of formulation variables on the baicalein release rate at 28 days for films F1–F9. Bars exceeding the reference line represent variables with a significant influence on drug release behavior.

In the next step, the release kinetics of baicalein from the developed chitosan-based films were evaluated. As shown in Figure 8 and Table 5, the dissolution profiles for all formulations (F1–F9) exhibited an excellent fit to zero-order kinetics, with R^2^ values consistently ranging from 0.98 to 0.99. This indicates a constant and controlled release rate of baicalein over time, independent of the remaining drug concentration within the matrix. The release rate constant (K) decreased systematically with both increasing chitosan concentration and molecular weight. Specifically, films containing low molecular weight chitosan (F1–F3) showed the highest release rates (e.g., K = 3.56 for F1), while those with high molecular weight chitosan and higher polymer content (F9: HMW, 3% CS) had the lowest (K = 1.87). These findings are corroborated by the Pareto chart (Figure 9), which statistically confirmed that both the chitosan concentration and its molecular weight significantly influenced baicalein release. Importantly, both factors had a negative effect: as either parameter increased, the percentage of baicalein released decreased. This phenomenon can be attributed to the formation of a denser and less permeable polymer network, which hinders drug diffusion [47]. Higher molecular weight chitosan contributes to increased viscosity and greater chain entanglement, while a higher concentration of chitosan results in tighter polymer packing [48]. Together, these properties reduce the mobility of the drug molecules, thereby slowing the release. This knowledge provides a valuable basis for tailoring film formulations according to therapeutic needs—favoring lower chitosan content and molecular weight for faster release or higher levels for sustained drug delivery.

**Figure 10:**
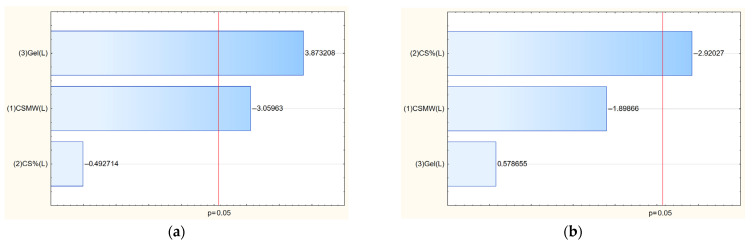
Pareto chart showing the standardized effects of antioxidant (**a**) and anti-inflammatory (**b**) activity of films 1–9.

The antioxidant and anti-inflammatory activities of baicalein-loaded chitosan films were analyzed based on IC_50_ values (Table 6) and statistically evaluated through Pareto charts (Figure 10). The antioxidant capacity, expressed as DPPH radical scavenging activity, varied significantly across formulations, with the lowest IC_50_ (i.e., strongest activity) observed for F8 (0.1283 mg film/mL) and F7 (0.1646 mg film/mL), while the weakest antioxidant effect was seen in F2 (0.5030 mg film/mL). The anti-inflammatory activity, evaluated via hyaluronidase inhibition, showed similar variability, with F9 and F8 demonstrating the highest potency (lowest IC_50_ values of 1.0826 and 1.1372 mg film/mL, respectively).

According to the Pareto analysis (Figure 10a), the antioxidant activity was significantly influenced by two factors: the gelatin content (with a negative effect) and the chitosan molecular weight (with a positive effect). This means that increasing gelatin content reduced antioxidant activity, likely due to the dilution of baicalein or interference with its molecular interaction potential. Conversely, higher molecular weight chitosan was associated with enhanced antioxidant performance. This may be attributed to a better film-forming structure that stabilizes baicalein or improves its availability at the surface where radical scavenging reactions occur.

For anti-inflammatory activity, the Pareto chart (Figure 10b) revealed that both chitosan concentration and chitosan molecular weight had a statistically significant and positive influence on activity, demonstrated by a decrease in IC_50_ values as these factors increased. This suggests that denser and more entangled chitosan networks may enhance the retention and presentation of baicalein in a form more favorable for enzyme inhibition. The improved activity may also be linked to the inherent bioactivity of chitosan, which can synergistically support the anti-inflammatory effects of baicalein [5]. Overall, the data emphasize the tunability of the film’s biological properties by adjusting polymer parameters, with specific combinations of chitosan concentration and molecular weight enhancing either antioxidant or anti-inflammatory performance depending on the therapeutic goal.

**Figure 11:**
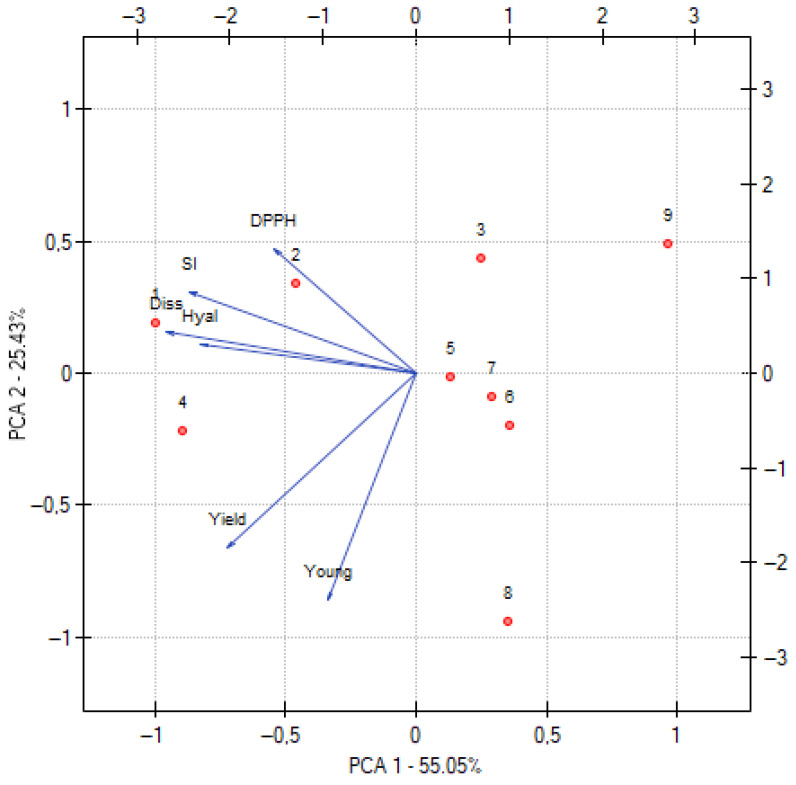
PCA analysis, where Young—Young modulus, Yield—yield stress, Diss—baicalein dissolution at 28 days, SI—swelling index, DPPH—antioxidant activity, Hial—anti-inflammatory activity.

The principal component analysis (PCA), illustrated in Figure 11, provides valuable insight into the interrelationships between the rheological, dissolution, and biological properties of the baicalein-loaded chitosan films. The matrix reveals several meaningful correlations. A strong positive correlation was observed between the swelling index (SI) and the dissolution rate (r = 0.938). This relationship suggests that formulations exhibiting higher swelling capacity tend to dissolve more efficiently. The likely mechanism behind this correlation is that increased swelling enhances the hydration of the matrix, leading to loosening of the polymer network. This structural relaxation allows for more effective penetration of dissolution medium, thereby accelerating the release of active compounds. Furthermore, greater water uptake increases the surface area available for diffusion, which contributes to improved dissolution kinetics. A strong positive correlation was observed between anti-inflammatory activity (Hial) and baicalein release (Diss) (r = 0.722), indicating that improved drug release enhances the ability of the films to inhibit hyaluronidase. A similar, though slightly weaker, positive relationship exists between antioxidant activity (DPPH) and dissolution (r = 0.536), suggesting that better baicalein availability also enhances radical scavenging effects. The anti-inflammatory activity further correlates positively with yield stress (r = 0.520), as does dissolution with yield stress (r = 0.607), implying that films with greater mechanical resistance may support sustained release and prolonged biological action. A very strong positive correlation was also observed between Young’s modulus and yield stress (r = 0.745), as expected given that stiffer materials tend to withstand greater stress before permanent deformation. In contrast, antioxidant activity (DPPH) showed virtually no correlation with mechanical parameters (r = 0.025 for Young and r = –0.002 for yield stress), indicating that its performance is largely independent of film stiffness or strength.

The PCA plot further clarifies these relationships by clustering variables with similar variance patterns. The first principal component appears to represent a mechanical–dissolution dimension, integrating Young’s modulus, yield stress, and drug release behavior. The second component likely captures variance in biological effects, especially antioxidant activity, which does not correlate strongly with mechanical traits. The positioning of DPPH, Hial, and Diss in the same quadrant highlights the central role of baicalein release in driving biological efficacy, particularly anti-inflammatory action. Meanwhile, antioxidant performance is more closely related to formulation variables—such as gelatin and chitosan characteristics—than to physical structure.

Overall, the PCA results reveal that the mechanical and biological attributes of the films can be tuned somewhat independently. While efficient baicalein release supports both antioxidant and anti-inflammatory activity, mechanical strength does not appear to limit biological performance. This multidimensional analysis underscores the potential for rational formulation design, allowing developers to optimize bioactivity without compromising structural integrity, or vice versa.

**Figure 12:**
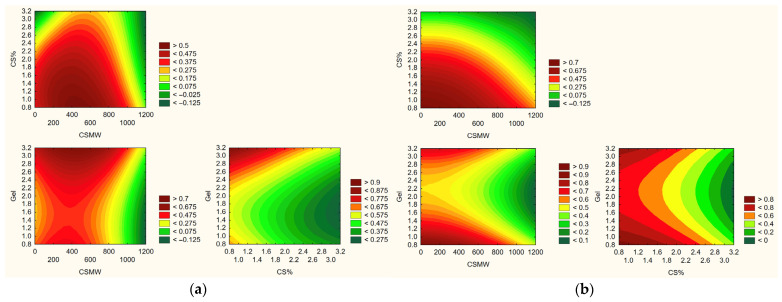
Model prediction for effects with a positive sign (release rate study) (**a**) and for effects with a negative sign (antioxidant and anti-inflammatory activity study) (**b**).

Based on the conducted research, a statistical model was predicted to select the optimal formulation composition (Figure 12).

According to the model, the optimal film composition is CS MMW 2% and Gel 2%, corresponding to system F5. This formulation represents a compromise between efficient baicalein release, strong antioxidant and anti-inflammatory activity, and favorable mechanical properties. F5, with its balanced composition, avoids these extremes. It provides sufficient matrix density for sustained release without overly hindering baicalein diffusion, while maintaining adequate biological activity. The statistical model integrates these findings and confirms F5 as the most robust and multifunctional formulation. Its positioning at the intersection of optimal release and bioactivity makes it particularly promising for clinical applications requiring both therapeutic efficacy and structural reliability, such as oral wound dressings or localized drug delivery systems.

Further studies were performed for formulation 5 to confirm further biological activity and clinical utility.

The microbiological evaluation (Table 7) of the developed baicalein-loaded chitosan film demonstrated promising antibacterial activity against *Streptococcus mutans*, a key pathogen involved in the development of dental caries and biofilm formation. During the assay, inhibition zones were clearly observed around the tested films, confirming their ability to suppress bacterial growth. The presence of well-defined inhibition zones indicates that baicalein retained its antimicrobial activity after incorporation into the chitosan–gelatin matrix and was effectively released in sufficient concentration to exert a bacteriostatic or bactericidal effect. This result is particularly important in the context of oral applications, as *S. mutans* is a major contributor to dental plaque accumulation, acid production, and subsequent enamel demineralization [49]. *Klebsiella pneumoniae* is a Gram-negative opportunistic bacterium associated with severe infections such as pneumonia, urinary tract infections, and periodontitis, particularly in immunocompromised individuals or patients with compromised oral hygiene. It has also been increasingly recognized as a contributor to biofilm formation on oral prosthetics and mucosal surfaces, where its resistance to conventional antibiotics poses a major therapeutic challenge [50]. Meanwhile, *Candida albicans* is the most common fungal species responsible for oral candidiasis and denture stomatitis, particularly in elderly or immunosuppressed patients. Its ability to adhere to epithelial surfaces, form biofilms, and switch between yeast and hyphal forms contributes to its pathogenicity and resistance to antifungal agents [51]. The inhibition of *C. albicans* by the tested film suggests potential use not only in antibacterial applications but also as a barrier against fungal colonization. The film’s ability to inhibit this microorganism directly supports their dental utility, especially in preventive and therapeutic strategies aimed at reducing microbial colonization in the oral cavity, managing localized infections following dental procedures, and serving as bioactive barrier membranes or post-extraction dressings with intrinsic antibacterial protection.

Moreover, the observed antibacterial effect complements the film’s antioxidant and anti-inflammatory properties, enhancing their potential as multifunctional biomaterials in periodontal therapy, caries prevention, and regenerative dentistry.

Biocompatibility is one of the key requirements for the successful application of membranes and scaffolds in regenerative medicine. An effective strategy to enhance the tissue-friendliness of film-based composites involves the use of inherently biocompatible polymers—such as chitosan and gelatin—and the careful selection of non-toxic, bioactive additives. The composition of the proposed scaffold meets these criteria, justifying its further biological evaluation. To confirm the safety of the material at the cellular level, cytotoxicity was assessed using the MTT assay, a widely accepted method for evaluating the biocompatibility of dental and biomedical materials [52]. The test was performed using a 1% concentration of the films in the culture medium, along with relevant concentrations of each component of the composite. No negative impact on the viability of fibroblasts was observed. These findings confirm the established biocompatibility of chitosan, which has been well-documented in the literature [53].

To assess the clinical applicability of the developed membranes in contact with biological fluids, behavioral testing was conducted using whole blood and injectable platelet-rich fibrin (iPRF), simulating surgical and regenerative conditions.

**Figure 13:**
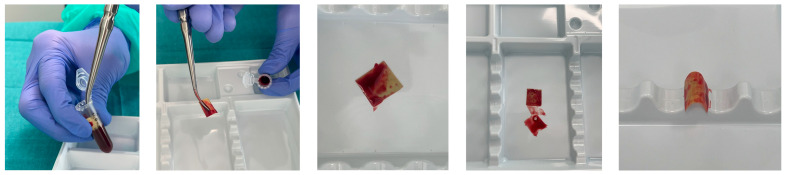
Blood-induced behavioral testing.

Evaluation of membrane adhesion and absorption (“blood uptake”) was performed after immersing the film in the venous blood. After a 5-min incubation period, the membrane was removed for visual inspection. Blood was observed adhering to the surface of the membrane but was not absorbed into its internal structure, as clearly seen in the images below. Upon removal from the tube, blood remained on the surface of the membrane; however, when the membrane was placed on a surgical tray, the blood detached and remained on the tray, demonstrating that it was not permanently bound to the material (Figure 13). This behavior may positively influence the clinical performance and application of the membrane. Its primary function is to act as a barrier separating the bone defect from surrounding soft tissues, while conforming closely to the shape of the underlying bone. Importantly, over time, the membrane did not revert to its flat form but rather maintained close and consistent contact with the cylindrical test surface, which mimics the shape of an alveolar ridge or bony contour. The observed partial interaction with blood—where blood adheres but is not fully absorbed—suggests that the membrane has favorable surface characteristics for surgical use. In clinical scenarios such as guided bone or tissue regeneration, barrier membranes should not behave like sponges. Excessive absorption could result in swelling, dimensional instability, or unwanted integration of blood clots into the membrane structure, which might interfere with the healing process [54]. Instead, an optimal membrane should interact superficially with blood, allowing for initial clot stabilization and biological signaling, without compromising structural integrity. The lack of deep blood penetration seen here aligns with properties expected from semi-permeable membranes used in bone regeneration: it allows for selective permeability, promotes clot retention at the bone surface, and helps maintain a protected space for osteogenic cell migration [55]. Furthermore, the membrane’s form-retaining behavior—demonstrated by its ability to maintain close contact with a curved surface—enhances its potential clinical utility. A membrane that conforms and stays in place over a defect area without springing back or lifting ensures better stability of the underlying graft or regenerating tissue. Literature supports that form stability is crucial for optimal bone fill and regeneration, particularly in non-self-supporting defects [56]. Lastly, the limited blood retention also implies a reduced risk of premature degradation in vivo, as excessive fluid absorption is associated with faster breakdown of natural polymers such as gelatin and chitosan. This balance between hydrophilicity and dimensional control reflects an advantageous combination for barrier function and handling during surgery.

**Figure 14:**
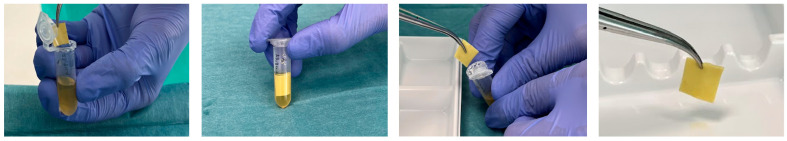
Verification of film adhesion and absorption—after immersion in iPRF.

Then, during testing of the membranes in contact with iPRF (injectable platelet-rich fibrin), their ability to bind and absorb platelet-rich plasma was evaluated (Figure 14). After immersion in iPRF, it was observed that the plasma adhered well to the surface of the membrane, forming a uniform layer. However, full absorption of the plasma into the internal structure of the material did not occur. Once the membrane was removed from the test tube and placed on a surgical metal tray, the iPRF remained on the tray surface rather than staying attached to the membrane. This indicates that the interaction between the membrane and iPRF was superficial and reversible, not involving permanent binding. This behavior may positively influence the clinical application of the tested membranes, especially in the context of their function as barrier materials in guided tissue regeneration and bone regeneration procedures. The primary role of such membranes is to isolate soft tissues from underlying bone and to support the regeneration process by stabilizing the defect area and maintaining space for new tissue growth. Chitosan-based biomaterials have shown promising interactions with platelet-rich plasma derivatives such as iPRF. According to multiple studies, chitosan can support the adhesion of growth factors and promote cellular activity due to its positively charged amino groups, which facilitate electrostatic interactions with negatively charged plasma proteins and components of iPRF [57]. However, despite this affinity, chitosan does not strongly absorb iPRF into its structure. Instead, it forms a temporary interface that allows for initial adhesion of the fibrin matrix and platelets, without sequestering them permanently [58]. Moreover, literature indicates that chitosan’s semi-hydrophilic nature plays a key role in this behavior. It enables the formation of a thin moisture film or gel layer on the surface without compromising the membrane’s dimensional stability [59]. This behavior is considered ideal for applications requiring controlled exposure to bioactive substances like iPRF, allowing gradual release of growth factors while maintaining the membrane’s barrier function. Additionally, some studies have highlighted the synergistic effects of combining chitosan membranes with PRF/iPRF, reporting enhanced angiogenesis, fibroblast migration, and osteoblast differentiation in vitro [60,61]. These effects stem not from deep absorption, but from the controlled presentation of bioactive molecules at the membrane–tissue interface.

**Figure 15:**
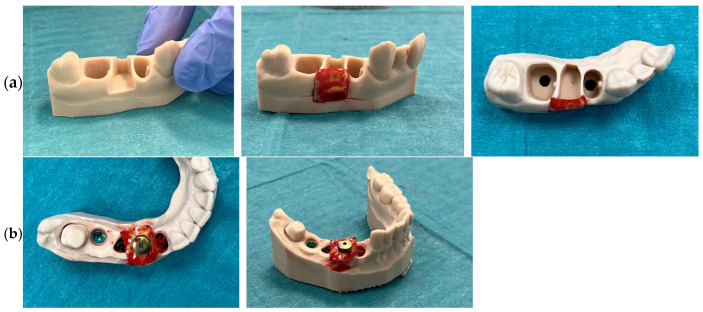
Research on 3D anatomical model = bone defect (**a**) and using implant analog (**b**).

The evaluation of a baicalein-loaded chitosan film on a 3D anatomical model with bone defect was also performed (Figure 15a). A study was conducted on a 3D-printed anatomical model simulating a single-walled buccal bone defect. A chitosan membrane enriched with baicalin and soaked in the patient’s own blood was applied to verify its adaptation, barrier properties, and ability to secure the bone defect. A deliberate space was left within the defect to prevent the membrane from resting on a potential graft material. In clinical practice, this membrane is intended to be placed between the bone graft and a collagen membrane. Owing to the properties of both baicalin and chitosan, the membrane provides slow release of active substances with antibacterial effects (as confirmed in the referenced study) and anti-inflammatory action, thus accelerating healing during the critical initial wound closure period. The membrane demonstrated excellent adaptation to the defect after mixing with venous blood from the patient.

On the printed 3D model, an implant analog corresponding to a clinical case was also positioned (Figure 15b). In the context of peri-implantitis treatment, after decontamination of the implant surface (mechanically, chemo-mechanically, and with laser assistance), bone augmentation and coverage with a PRF membrane are typically performed. The chitosan–baicalin membrane showed promise when applied at the junction between the healing abutment and the implant, drawing an analogy to the “punch technique” with a PRF membrane alone. For this procedure, two perpendicular 5 mm incisions are made with a scalpel, creating a cross-shaped opening to accommodate the abutment or prosthetic component. The membrane soaked in patient blood can be easily trimmed without tearing, significantly simplifying clinical handling. Cutting this membrane proved easier than working with membranes soaked in azithromycin. The positioning of the abutment together with the membrane was optimal, allowing for precise and comfortable clinical work. Thanks to the thin profile of the membrane, an additional layer of aPRF can be placed to further stimulate soft tissue regeneration. For the purposes of this study, this layered construct may be referred to as a “sandwich system”: augmented bone at the implant site + baicalin–chitosan membrane + aPRF membrane + wide healing abutment head (wider than the implant platform), providing extra protection. The entire assembly remained stable due to the secure connection with the implant. The membrane demonstrated both plasticity and shape retention, and during abutment placement, it did not tear or deform, as confirmed in photographic documentation. This enabled uniform distribution of the membrane around the bone defect.

An additional clinical advantage is the membrane’s color. Baicalin imparts a slightly yellowish hue, converting the otherwise transparent chitosan into a more matte appearance. This is significant in the surgical field where procedures are performed under conditions of flap elevation and bleeding. A transparent membrane in contact with bone or mucosa can easily be lost in the surgical field, especially when it adheres to oral structures. Furthermore, in procedures requiring constant suction of blood and tissue debris, a transparent membrane could be inadvertently aspirated by the assistant. The distinctive color of the baicalin–chitosan membrane reduces this risk and enhances visibility during surgery.

**Figure 16:**
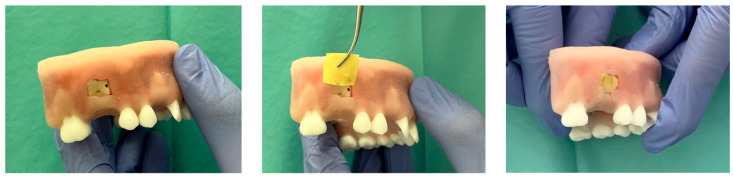
Evaluation of a material in a 3D anatomical model of soft tissue.

Finally, evaluation of a baicalein-loaded chitosan film in a 3D anatomical model of soft tissue for donor site protection following free gingival graft harvesting (Figure 16). One of the most performed procedures in periodontal surgery is the use of a free gingival graft (FGG) to cover gingival recessions. This technique involves harvesting tissue from the palate and transplanting it to a site requiring an increase in the width of keratinized gingiva and augmentation of soft tissue volume. The procedure improves aesthetics and facilitates oral hygiene, contributing to the long-term survival of dental implants or natural teeth. Harvesting the graft leaves a donor site wound on the palate, typically secured with sutures (and optionally covered with a PRF membrane), which takes several weeks to heal. During this healing process, patients often report discomfort, pain, swelling, and redness. Moreover, when clinical conditions require additional procedures—such as covering multiple recessions in other parts of the oral cavity—patients are frequently discouraged from proceeding with further treatments due to their previous experience of postoperative discomfort, which complicates the completion of comprehensive treatment plans. In this study, conducted on a 3D anatomical model simulating the clinical situation (including both soft and hard tissues), the donor site was covered with a baicalein-loaded chitosan film. The antibacterial and healing-promoting properties of the membrane are expected to enhance the healing process at the donor site while providing physical protection to the wound. The membrane used consisted of chitosan enriched with baicalin, pre-soaked in a 0.9% NaCl solution. This soaking aimed to soften the membrane structure, facilitating better adaptation to the donor site and enabling precise trimming of the membrane after swelling (ensuring that no material extended beyond the wound margins). Due to the inability to secure the membrane directly to the gingiva with sutures—since attempts resulted in tearing of the material—a mattress suture was employed. This suture served solely to approximate the wound edges and provide mechanical stabilization of the membrane over the donor site. In real clinical conditions, the membrane would additionally be stabilized by the natural blood clot forming over the wound, further securing it in place.

## 4. Conclusions

This study provides a comprehensive evaluation of baicalein-loaded chitosan–gelatin films, highlighting their potential as multifunctional biomaterials for oral and maxillofacial applications. The films, developed through a 3^2^ factorial design, demonstrated that chitosan molecular weight and formulation composition significantly influence mechanical properties, drug release kinetics, and biological activities.

All tested systems achieved uniform baicalein dispersion and followed zero-order release kinetics, supporting controlled and sustained delivery. Medium molecular weight chitosan formulations, particularly F5 (CS MMW 2%, Gel 2%), offered an optimal balance of mechanical strength, flexibility, sustained release, and biological efficacy. The F5 formulation exhibited excellent antioxidant and anti-inflammatory activity, confirmed by statistically significant correlations between release rate and bioactivity.

Beyond physicochemical and in vitro biological characterization, the films were evaluated in clinically relevant simulations. On 3D anatomical models, the membranes demonstrated excellent adaptability and barrier function in both bone defects and soft tissue donor site scenarios. In bone defect models, the films conformed well to irregular surfaces, maintained structural integrity during placement, and supported a layered regenerative approach (“sandwich system”) with PRF. The distinctive coloration of the baicalein-loaded membrane enhanced surgical visibility, addressing a practical challenge of membrane handling under conditions of bleeding and limited visibility. In soft tissue models, the membrane provided physical protection to donor sites after free gingival graft harvesting, with handling characteristics that facilitate clinical use. Its partial interaction with blood and iPRF suggests a favorable balance between surface adhesion and dimensional stability, key features for successful regenerative outcomes.

The film’s safety profile was confirmed through cytocompatibility testing, and antibacterial activity against *Streptococcus mutans* further supports its potential utility in managing oral infections and promoting healing. The modular nature of the system also allows for future integration of additional bioactive agents tailored to specific clinical needs.

In summary, baicalein-loaded chitosan films represent a promising class of multifunctional, bioactive membranes for targeted, localized therapy in oral surgery, periodontal regeneration, and peri-implant care. Future in vivo studies and clinical trials are warranted to confirm these findings and support regulatory approval for clinical application.

## Figures and Tables

**Table 1:** Composition of chitosan films.

	CS MW	CS %	Gel %
F1	100	1	1
F2	100	2	3
F3	100	3	2
F4	600	1	3
F5	600	2	2
F6	600	3	1
F7	1100	1	2
F8	1100	2	1
F9	1100	3	3

**Table 2:** Baicalein bands (in cm^−1^) for which changes are observed in the F1–F9 (black—decrease in intensity, blue—peak shift).

	Baicalein Peaks (cm^−1^)
F1–F3	424, 461, 488, 523, 598, 638, 681, 733, 777, 826, 853, 897, 916, 1020, 1159, 1331
F4–F6	488, 523, 571, 598, 615, 638, 681, 706, 733, 777, 826, 853, 897, 916, 1020, 1032, 1103, 1159, 1331, 1449
F7–F9	488, 523, 571, 598, 615, 638, 681, 706, 733, 777, 826, 853, 897, 916, 1020, 1032, 1103, 1159, 1331, 1449

**Table 3:** Gelatin bands (in cm^−1^) for which peak shifts were observed in samples F1–F9.

GEL	F1	F2	F3	F4	F5	F6	F7	F8	F9	Assignment
1234	1240			1240			1238			ν_C-N_ + δ_NH_ (Amide III)
1393	1391			1396	1389	1398	1391	1381	1391	$\omega_{{\mathrm{CH}}_{2}}$ of proline
1445	1452			1452	1449	-	1449			$\delta_{{\mathrm{CH}}_{2}}$
1524	1551			1551			1553	1553	1549	ν_C-N_ + ν_C-C_ + δ_NH_ (Amide II)
1634	1641			1641	1645	1649	1641	1651	1643	ν_C=O_ of carboxylate anion + ν_NH_ (Amide I)

**Table 4:** Young’s modulus and yield stress values for films F1–F9.

Strain Rate	0.005 1/s		0.02 1/s		0.05 1/s		0.005 1/s	0.02 1/s	0.05 1/s
	Young’s Modulus [kPa]	R^2^	Young’s Modulus [kPa]	R^2^	Young’s Modulus [kPa]	R^2^	Yield Stress [kPa]	Yield Stress [kPa]	Yield Stress [kPa]
F1	60.07	0.99	119.74	0.99	125.67	0.99	3134.90	3060.70	3042.30
F2	119.56	1.00	140.27	0.99	133.80	0.99	2265.30	2260.10	2259.20
F3	89.00	1.00	107.08	1.00	102.24	1.00	1891.60	1885.60	1885.00
F4	105.99	0.98	166.87	1.00	161.79	0.99	3041.60	3024.30	3021.80
F5	112.59	1.00	131.16	0.99	131.15	0.99	2267.10	2261.50	2259.70
F6	133.58	1.00	144.43	0.98	143.44	0.99	2256.30	2252.80	2252.50
F7	82.82	0.96	127.34	0.99	122.20	0.99	2278.50	2272.00	2269.70
F8	139.39	0.99	176.80	0.99	175.40	0.99	3031.60	3012.10	3009.60
F9	85.93	1.00	94.54	0.99	90.07	0.99	1505.00	1503.30	1503.50

**Table 5:** Parameters of mathematical models fitted to the baicalein release profiles from films F1–F9.

	Mathematical Model							
No.	Zero-Order Kinetic		First-Order Kinetic		Higuchi Kinetic		Korsmeyer–Peppas Kinetic	
	K	R^2^	K	R^2^	K	R^2^	R^2^	n
F1	**3.56**	**0.98**	0.19	0.62	6.29	0.70	0.84	0.47
F2	**3.13**	**0.99**	0.18	0.62	5.45	0.68	0.88	0.45
F3	**2.22**	**0.99**	0.20	0.51	3.79	0.66	0.72	0.50
F4	**2.99**	**0.99**	0.20	0.56	5.14	0.67	0.80	0.50
F5	**2.53**	**0.99**	0.20	0.58	4.42	0.69	0.78	0.48
F6	**2.13**	**0.99**	0.20	0.58	3.68	0.67	0.75	0.46
F7	**2.32**	**0.99**	0.20	0.61	3.92	0.65	0.74	0.46
F8	**2.13**	**0.99**	0.19	0.59	3.66	0.67	0.78	0.46
F9	**1.87**	**0.99**	0.21	0.58	3.15	0.64	0.71	0.47

The best fit to the model is bolded.

**Table 6:** Biological activity results.

	Antioxidant Activity Inhibition of DPPH Radical Activity IC_50_ [mg film/mL]	Anti-Inflammatory Activity Inhibition of Hyaluronidase Enzyme Activity IC_50_ [mg film/mL]
F1	0.2697 ± 0.0097 ^d^	3.0199 ± 0.2880 ^f^
F2	0.5030 ± 0.0148 ^f^	1.8936 ± 0.0707 ^c^
F3	0.2970 ± 0.0043 ^d^	2.2718 ± 0.1011 ^d,e^
F4	0.4590 ± 0.0369 ^e^	3.6246 ± 0.1455 ^g^
F5	0.2051 ± 0.0222 ^c^	2.1007 ± 0.0194 ^c,d^
F6	0.2306 ± 0.0102 ^c^	1.6675 ± 0.0559 ^b^
F7	0.1646 ± 0.0269 ^b^	2.3631 ± 0.1382 ^e^
F8	0.1283 ± 0.0109 ^a^	1.1372 ± 0.0407 ^a^
F9	0.2868 ± 0.0065 ^d^	1.0826 ± 0.0647 ^a^
Baicalein	0.0921 ± 0.0071 mg/mL	1.9848 ± 0.0140 mg/mL

Mean values in a column marked with the same letter are not statistically significantly different at *p* = 0.05, according to Duncan’s test.

**Table 7:** Growth inhibition zone values (in mm).

	*Staphylococcus aureus*	*Klebsiella pneumoniae*	*Candida albicans*
Inhibition zone [mm]	5	9	5

## Data Availability

Data are contained within the presented article.
